# Black Petrels (*Procellaria parkinsoni*) Patrol the Ocean Shelf-Break: GPS Tracking of a Vulnerable Procellariiform Seabird

**DOI:** 10.1371/journal.pone.0009236

**Published:** 2010-02-17

**Authors:** Robin Freeman, Todd Dennis, Todd Landers, David Thompson, Elizabeth Bell, Mike Walker, Tim Guilford

**Affiliations:** 1 Computational Ecology and Environmental Science, Microsoft Research, Cambridge, United Kingdom; 2 Animal Behaviour Research Group, Department of Zoology, University of Oxford, Oxford, United Kingdom; 3 School of Biological Sciences, University of Auckland, Auckland, New Zealand; 4 Auckland War Memorial Museum, Auckland, New Zealand; 5 National Institute of Water and Atmospheric Research Ltd, Kilbirnie, Wellington, New Zealand; 6 Wildlife Management International Limited, Rapaura, Blenheim, New Zealand; Institut Pluridisciplinaire Hubert Curien, France

## Abstract

**Background:**

Determining the foraging movements of pelagic seabirds is fundamental for their conservation. However, the vulnerability and elusive lifestyles of these animals have made them notoriously difficult to study. Recent developments in satellite telemetry have enabled tracking of smaller seabirds during foraging excursions.

**Methodology/Principal Findings:**

Here, we report the first successful precision tracking of a *c*. 700 g seabird, the vulnerable Black Petrel, *Procellaria parkinsoni,* foraging at sea during the breeding season, using miniature GPS-logging technology. Employing a combination of high-resolution fixes and low-power duty-cycles, we present data from nine individual foraging excursions tracked during the chick-rearing period in February 2006.

**Conclusions/Significance:**

We provide a snapshot of the species' foraging range and behaviour in relation to detailed underlying bathymetry off the coast of New Zealand, finding a significant relationship between foraging movements and regions of the shelf-break. We also highlight the potential of more sophisticated analyses to identify behavioural phenomena from position data alone.

## Introduction

The oceanic lifestyles of pelagic seabirds are amongst the most elusive on Earth. Recently, an awareness of the importance of pelagic seabirds as integrators of global marine resources (and hence indicators of ocean health in an age of accelerating anthropogenic environmental change), has given momentum to attempts to understand their extraordinary lifestyles and the threats posed to them. The need to identify accurately essential foraging habitats, locations of incidental mortality, or at-sea aggregations requires high-resolution tracking information. Studies using Platform Terminal Telemetry (PTT) have shown the remarkable foraging patterns of albatross species and larger petrels [1] and smaller technologies, such as light-logging geolocators [2], have been employed with smaller pelagic species to record detailed global migrations for the first time [3], [4], [5]. However, these systems still suffer from a lack of spatial resolution [6]. GPS tracking can provide a much greater spatial resolution, to within a few metres, and has been successfully used in a variety of studies such as foraging albatrosses [7], [8], [9], Shearwaters [10], Gannets [11], [12] and Penguins [13], [14]. A combination of this high spatial resolution and large quantities of data can also allow the application of methods traditionally limited to data-rich fields like machine learning [15], [16]. However, until recently, weight-constraints have meant that GPS tracking studies have been limited to these larger species or to shorter deployments.

The Black Petrel *(Procellaria parkinsoni)* is listed as vulnerable (VU D2 on the IUCN Red List; [17]) with only around 2000 breeding pairs remaining which are restricted to the islands of Great Barrier and Little Barrier, New Zealand. Their restricted habitat, predation from feral cats and rats and the uncertain impacts from long-line fishing have all contributed to their vulnerability [18], [19]. Black Petrels are specifically covered by the International Agreement on the Conservation of Albatrosses and Petrels (2001) which obliges signatories to reduce incidental mortality, control detrimental non-native species, protect critical habitats and support research into the highlighted species [20].

Black Petrels forage widely at sea, migrating to waters of the eastern tropical pacific in the austral winter where they have been recorded moving in groups with cetaceans and scavenging scraps from the cetaceans' feeding [21]. They breed underground, visiting their remotely-situated colony only at night, so they are especially resistant to normal methods of study. Consequently, they are little understood despite their important conservation status. However, because Black Petrels faithfully return to their nesting burrows, where they are relatively easy to capture repeatedly, they lend themselves to the use of lightweight GPS-based logging devices first developed for use with homing pigeons [22], [23]. By using miniature GPS technology, low-power tracking techniques and modern analytical methods, we present here the successful tracking and analysis of foraging 700 g Black Petrels from Great Barrier Island, New Zealand. We present data from 9 individual foraging excursions tracked during the chick-rearing period in February 2006, providing a first snapshot of this vulnerable species' foraging range and behaviour in relation to detailed underlying bathymetry.

## Methods

The network of previously established study burrows in the breeding colony on Mount Hobson (Hirakimata), GBI, was used during the study. This network was created and maintained by NZ Department of Conservation and Wildlife Management International Limited over the previous ten years. From 22^nd^ February to 9^th^ March 2006, 14 GPS logging devices were deployed for the duration of single foraging trips on breeding birds parenting chicks. Entrances to burrows of known breeding pairs were marked with small sticks to detect entry or exit, and were inspected at 30-minute intervals throughout each night. When a bird arrived at its burrow it was allowed to feed its chick, was then removed manually, weighed and, if of suitable mass (>560 g), fitted with a GPS logging device, and returned to the burrow entrance. Devices were attached to feathers on the bird's back, above the centre of gravity, using four (occasionally five) 1-cm strips of waterproof cloth tape [10]. Devices were retrieved by re-capturing the birds after their return from foraging trips, and then peeling off the tapes. In most cases, birds were weighed again after deployment, having been given time to feed their chick.

The tracking devices consisted of a 12-channel GPS receiver with flash memory and built-in passive ceramic aerial (produced by μ-blox AG), and a 550 mAh 3.7 V lithium-polymer rechargeable battery (Allbatteries inc.), encased in the field in lightweight heat-shrink plastic tubing. In total devices weighed approximately 32 g including attachments, between 3.3% and 5% of body mass. Most of the devices were configured to maximise coverage of longer trips, and thus were programmed to take a short burst of position fixes every two hours. These short bursts allowed us to calculate accurately the speed within each ‘cluster’. Two additional devices were configured to take continuous (5-second interval) fixes once an initial fix was established, in order to obtain detailed data on flight behaviour for a shorter period. Data were downloaded to laptop computer after retrieval of the device and initially filtered to remove lower accuracy fixes that relied on fewer than 4 satellites (retaining so-called ‘3D’ fixes and higher), and some artefactual 0 ms^−1^ speeds.

Distributions of speeds were modelled using a mixture of Gaussians. We assumed that the observed data could be predicted by some underlying number of Gaussian probability distributions. The mean and variance of these distributions were iteratively fitted to the observed data (here we use expectation-maximisation; see [24]), and this model was then used to predict and classify the remaining data. Data were classified by assignment to the distribution from which they were most likely to have been generated. In the results reported here, all fitting and classifications were conducted using a model fitted to log-transformed data, to avoid assigning positive probabilities to speeds of less than zero, but the model is presented below on untransformed data for clarity. This method provides a more robust classification of behaviour that otherwise may have been biased by different GPS acquisition probabilities. In the low-resolution tracks, acquisition success rates may have been higher during particular behaviours (sitting, for example). However, by basing our movement model on near-complete tracks at high resolution, we hoped to mediate against such potential biases. This procedure may be of general applicability to tracking data where many of the samples may be sparsely collected or biased.

Bathymetric data were supplied by the New Zealand National Institute of Water and Atmospheric Research (http://www.niwa.gov.nz), and Chlorophyll *a* data in mg/m^3^ concentrations (Aqua MODIS at 9 km cell resolution) for the period of the study (one month averaged data for 15^th^ February to 16^th^ March) were downloaded from http://oceancolor.gsfc.nasa.gov.

## Results

Of the 14 devices we deployed, ten were recovered, nine with data. The remaining four birds returned without devices and all birds returned at approximately the same mass as at deployment (where measured). Table 1 summarises the deployments, and shows the weight of birds, measured at times of deployment and recovery of trackers. As the birds were weighed after they had completed feeding their chicks, any weight gains and small decreases observed imply that adults must have foraged successfully during deployment. While we cannot be certain that the birds gathered as much food as they could have had they not been carrying tracking devices, this does indicate that the tracks recorded include successful foraging behaviour.

**Table 1:** Deployment and Recovery information.

Ring #	Burrow #	Deployment	Recovery	Days	Mass at deployment (g)	Mass at recovery (g)
25459	144	26^th^ February	2nd March	4	900	850
25659	101	2nd March	4th March	2	700	660
25421	75	2nd March	3rd March	1	855	785
25507	140	23^rd^ February	6th March	11	600	660
30854*	7	24^th^ February	8th March	12	680	-
31155*	81	26^th^ February	Tracker Lost	-	585	-
31103	8	23^rd^ February	1st March	6	700	740
31272	7	24^th^ February	1st March	5	850	770
32005	68	2nd March	Returned Later	-	665	-
33312*	265	24^th^ February	Tracker Lost	-	580	-
33715	316	23^rd^ February	25th February	2	660	795
33757	244	24^th^ February	28th February	4	660	790
33759*	257	23^rd^ February	Tracker Lost	-	610	-
34999*	204	24^th^ February	Tracker Lost	-	690	-

Deployment and recovery of GPS trackers, and bird mass. Starred records indicate unsuccessful deployments, where the trackers was either lost, or recovered with no data. ‘Returned later’ indicates a device that was recovered from a bird subsequent to fieldwork where the exact recovery date is unknown.

**Figure 1:**
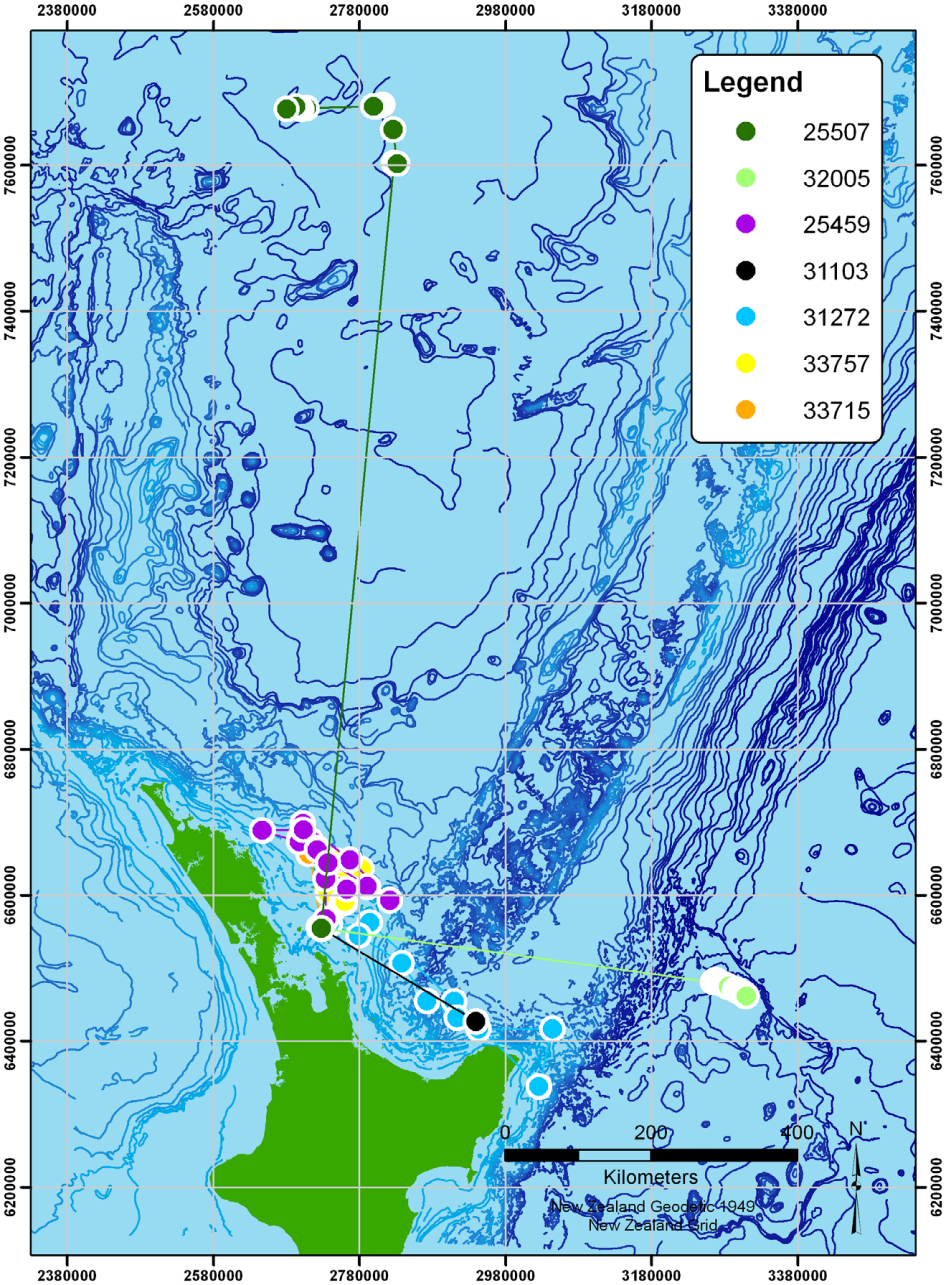
All Black Petrel tracks recorded using the low-resolution tracking method, in which clusters of fixes are recorded every two hours. Each bird is presented separately, with clusters joined by lines for clarity.

**Figure 2:**
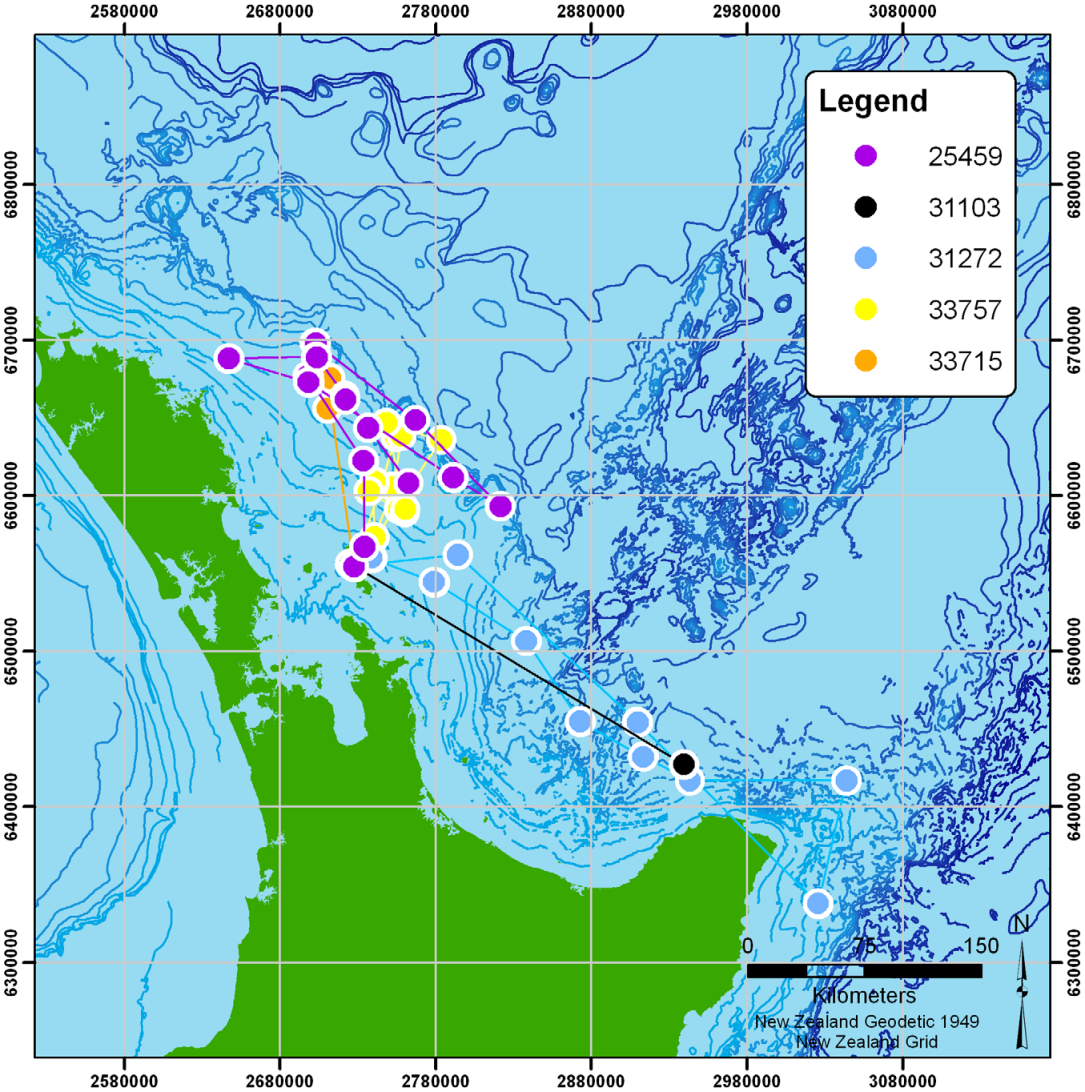
Local low-resolution tracks. Clusters are joined with lines for clarity.

**Figure 3:**
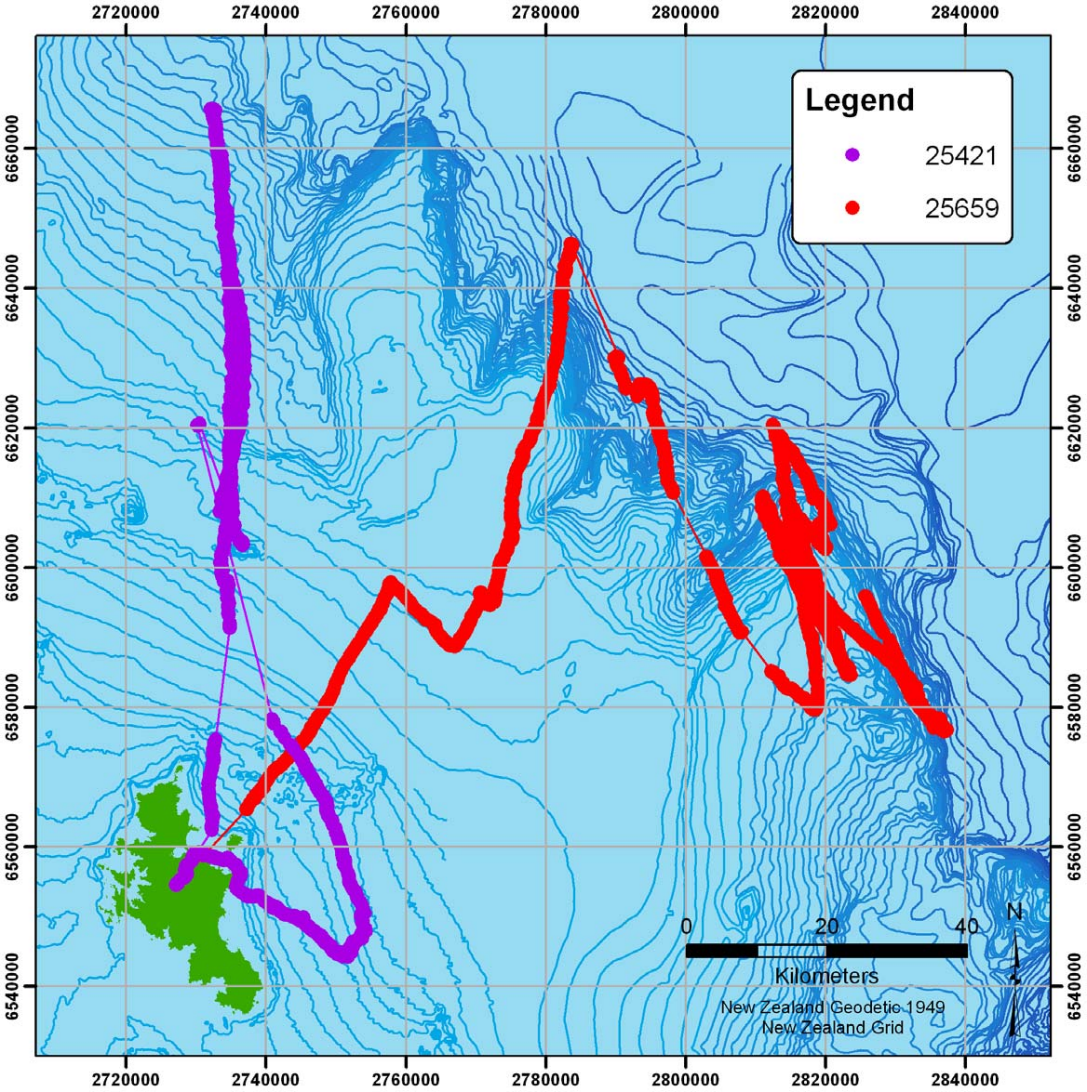
Two high-resolution (5 second interval) tracks. Regions where fixes were lost are joined by lines for clarity.

Figure 1 shows the seven two-hourly logged tracks. Each bird is indicated by a different colour, with clusters of fixes joined by lines to indicate the birds' progress. Two of the birds travelled substantially further than the rest (one reaching at least 1128 km from the colony, the other around 522 km). The more locally-foraging birds' tracks are also shown separately in figure 2. Figure 3 shows the two tracks recorded at high-resolution (5 second interval). Summary statistics of each track are given in table 2.

**Table 2:** Summaries of the nine successful deployments.

Ring #	Area (km^2^)	Furthest Point (km)	Length (km)
25459	4,676,390	156.12	746.35
25507	84,915,508	1128.14	2396.59
25421	552,622	111.1	367.77
25659	1,983,459	111.92	612.28
31103	9,433	247.87	496.21
31272	161,131	369.17	842.13
32005	2,588,387	552.43	1122.09
33715	100,432	122.38	248.39
33757	1,462,062	98.27	428.45

Each deployment shows the tracked bird (ring number given), the total area within the tracked route and the furthest point from the colony.

**Figure 4:**
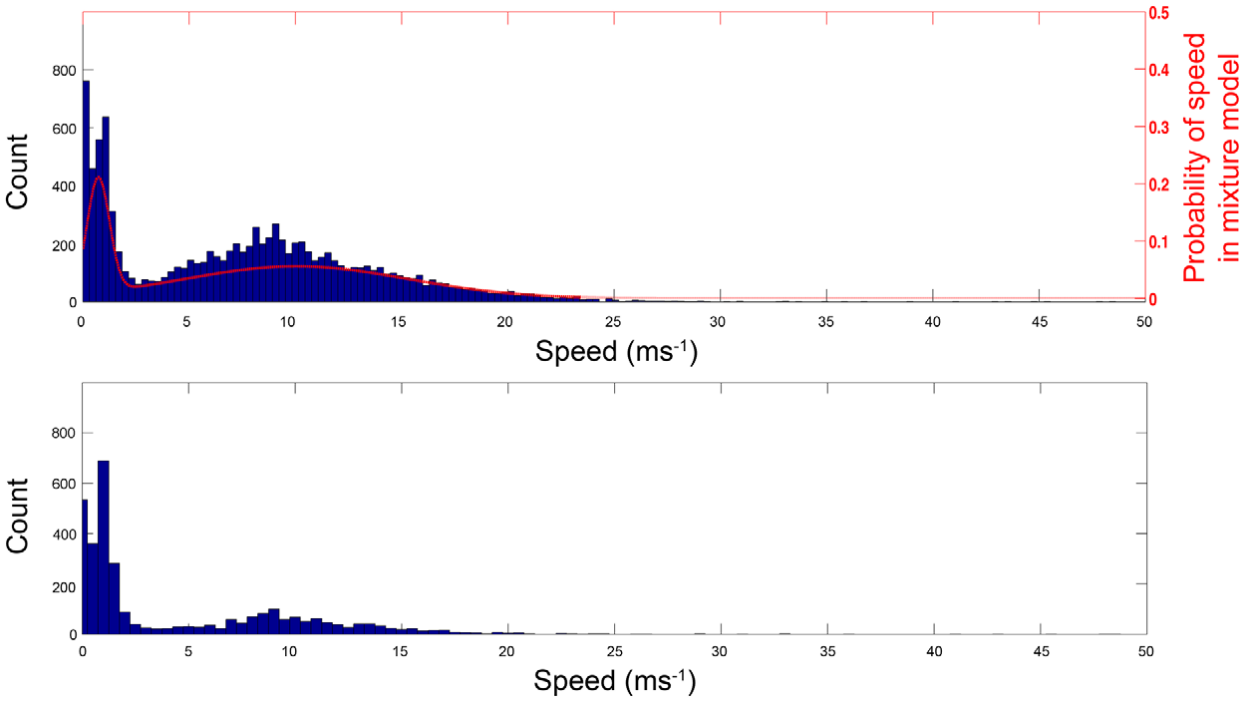
Histograms of speeds from high-resolution tracks (top) and low-resolution tracks (bottom). The probability of a given speed under the calculated model is shown by the red line (top, right-axis), total model probability sums to one.

We used the higher-resolution (5 second interval) tracks to provide detailed models of the speed of petrels at sea. Figure 4 (top, bars) shows a histogram of speeds within the high-resolution tracks. The distributions clearly show the existence of different types of movement: we hypothesise that the faster distribution represents flight, while the slower distribution represents sitting on the ocean surface and drifting with the current [10]. We fitted a Gaussian mixture model to these data to provide an estimate of mean speed for each type of movement (Slow peak: mean speed = 0.83 ms^−1^, variance 0.13 ms^−1^; Fast peak: mean speed = 10.20 ms^−1^, variance 27.0 ms^−1^). Figure 4 (top, red line) shows this model overlaid on the tracked speeds.

Using the values obtained from the mixture model fitted to the high-resolution data, we classified the clusters of data at each two-hour interval in the lower-resolution tracks. Each cluster of data can be assigned to the distribution from which it is most likely to have originated, giving a probability for each cluster as either ‘sitting’ (slow peak) or ‘flying’ (fast peak). The distribution of surface speeds for the lower-resolution tracks is broadly similar to the high-resolution tracks as shown in figure 4 (bottom vs. top). Fitting a mixture model to the low-resolution tracks also gives an estimate of movement speeds (Slow peak: mean speed = 0.97 ms^−1^, variance = 0.16 ms^−1^; Fast peak: mean speed = 9.54 ms^−1^, variance = 27.84 ms^−1^; N = 2703).

Table 3 shows, for each low-resolution track, the proportion of fixes in each category once classified.

**Table 3:** Classification of tracks.

Ring #	Slow Class (%)	Fast Class (%)
25459	18	82
25507	90	10
25421*	83	17
25659*	71	29
31103	100	0
31272	16	84
32005	17	83
33715	16	84
33757	93	7

Percentage of locations classified into the two groups, slow and fast, for each successfully tracked individual. Starred birds are tracked using the high-resolution method and can also be classified using the model.

### Relationship with Bathymetry

In those birds with sufficient fixes in both classes (N = 7), examination of the depth of the sea floor under each fix shows that in most individuals (6 out of 7) the distribution of depths in the slow class differed significantly from those in the fast class (Two-sample Kolmogorov-Smirnov goodness-of-fit tests, p<0.01, D = 0.2–0.84).

To generate a null distribution of the possible depths over which each bird could have flown, each track was rotated around its origin through fifteen 22.5° angles (fifteen equally spaced rotations through 360°). This produces a randomised sample of depths within the area that each bird could have utilised while maintaining the internal structure of the track itself. After excluding locations that were over land, the depth of the sea floor at those locations was recorded. Repeatedly comparing equally sized random distributions to those from the high-resolution and local low-resolution tracks shows a significant difference in all cases (p<0.05, Two-sample Kolmogorov-Smirnov goodness-of-fit, 1000 runs). Additionally subdividing the depths in each individual into those under the ‘slow’ and ‘fast’ classes also showed a significant difference between the depths under the randomised positions of those ‘slow’ and ‘fast’ locations (p<0.05, Two-sample Kolmogorov-Smirnov goodness-of-fit, 1000 runs).

Examining each track individually avoids potential biases caused by particular individuals, but pseudo-replication within the track may still have an effect. However, we note that with fixes recorded every 5 seconds, an individual may fly around 500 m (±250 m, using the measured speeds above) over 10 fixes, enough to see significant changes in the depth over which they are flying. For example, the band of 600–1000 m depths described below is as narrow as 700 m at some points. As such, the decision to remain over particular depths is important to identify, as it may be indicative of important bathymetric regions for the birds.

However, we can examine the two high-resolution tracks as a series of merged locations every two minutes. Here, the tracks are decomposed into two-minute windows with all fixes within a window merged into a single location at the window centre. In these birds, the comparisons described above remain significant: recorded depths under tracks differ from randomly sampled depths (p<0.05, Two-sample Kolmogorov-Smirnov goodness-of-fit, 1000 runs), depths under fast locations differ from those under slow locations (25421: p<0.01, *D* = 0.24; 25659: *p*<0.001, *D* = 0.23) and both of these subsets also differ from randomised samples (p<0.05, Two-sample Kolmogorov-Smirnov goodness-of-fit, 1000 runs).

**Figure 5:**
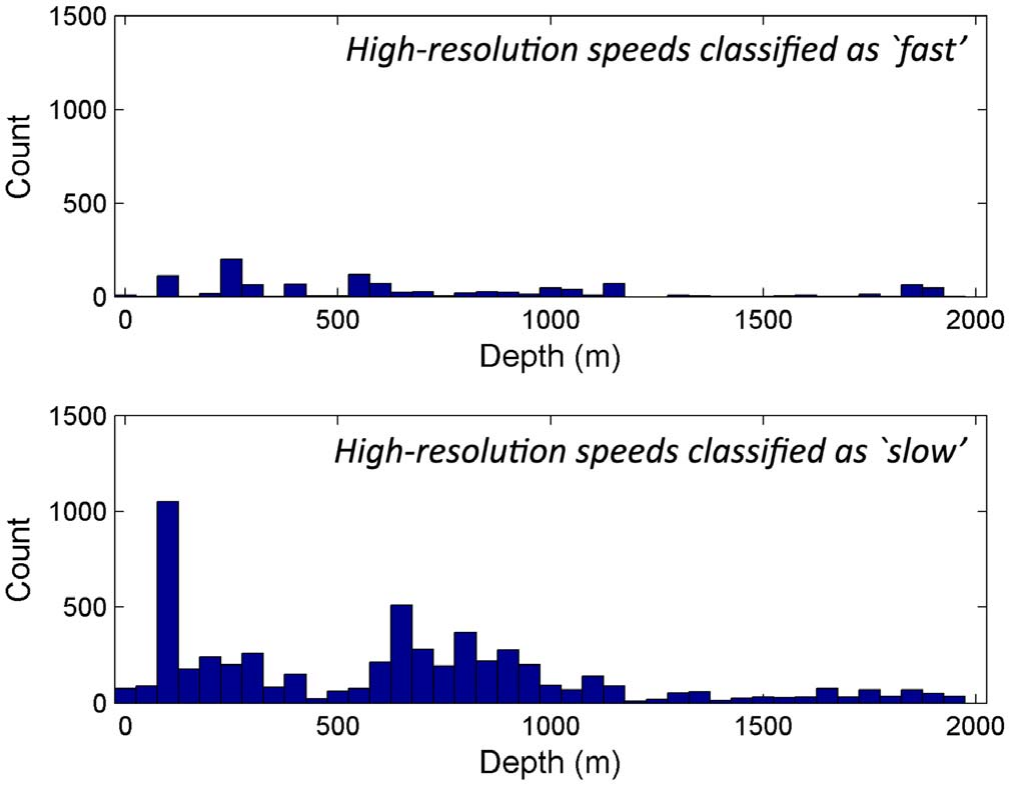
Depth under high-resolution tracks at positions classified as ‘slow’ or ‘fast’.

**Figure 6:**
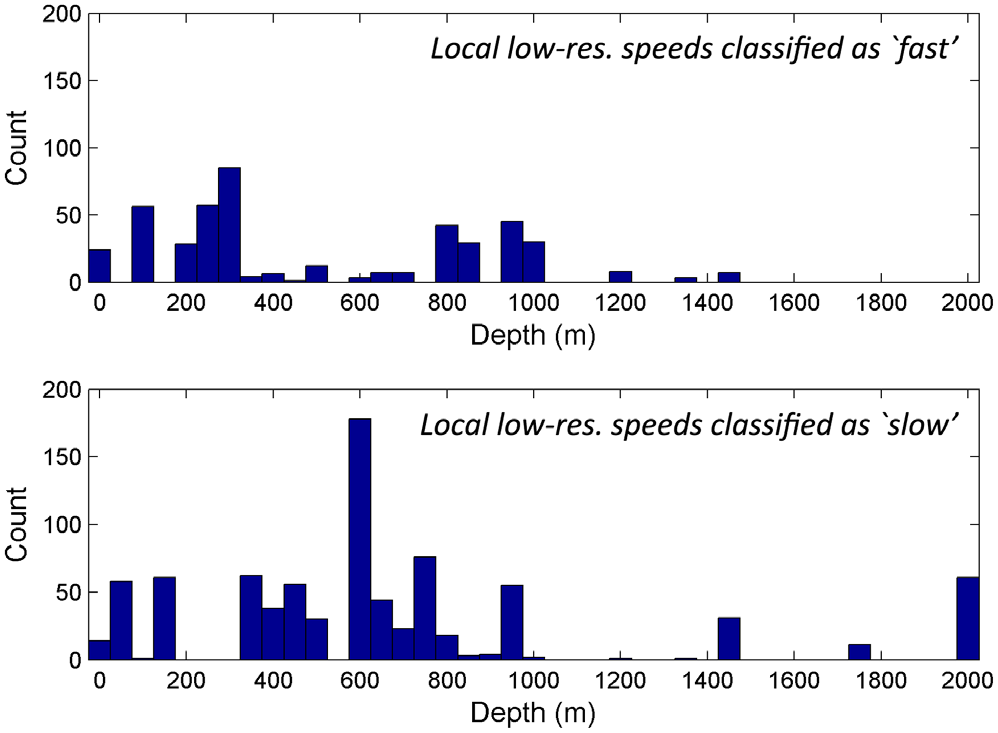
Depth under local, low-resolution tracks at clusters classified as ‘slow’ or ‘fast’.

Examining the distribution of depths in the slow class highlights the greater depths that the far-ranging birds (25507, 32005) utilise (∼2000 m), in comparison to the shallower (<2000 m) depths of the locally foraging birds. After removing the far-ranging birds, the remaining data can be further separated into high-resolution and low-resolution birds (figure 5 & figure 6 respectively).

**Figure 7:**
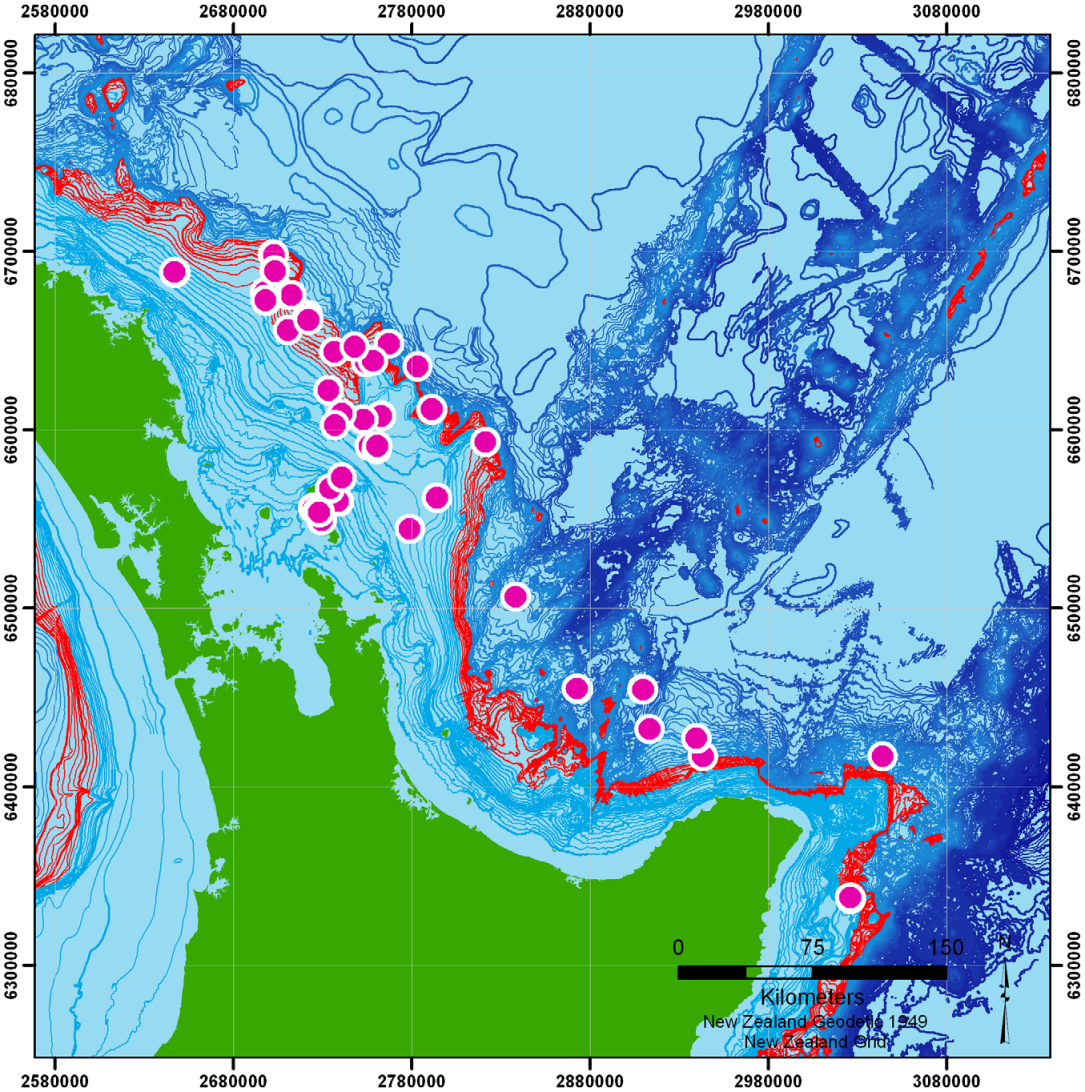
Locations overlaid on bathymetry with −600 m to −1000 m shelf-break marked (red). Pink circles indicate bird locations.

We also note that in both the high- and low-resolution tracks, the overall ‘slow’ distribution appears to show an increased likelihood for the birds to be recorded over depths of 600m–1000 m. Figure 7 shows the local positions overlaid on depth contours for the region with this area highlighted.

**Figure 8:**
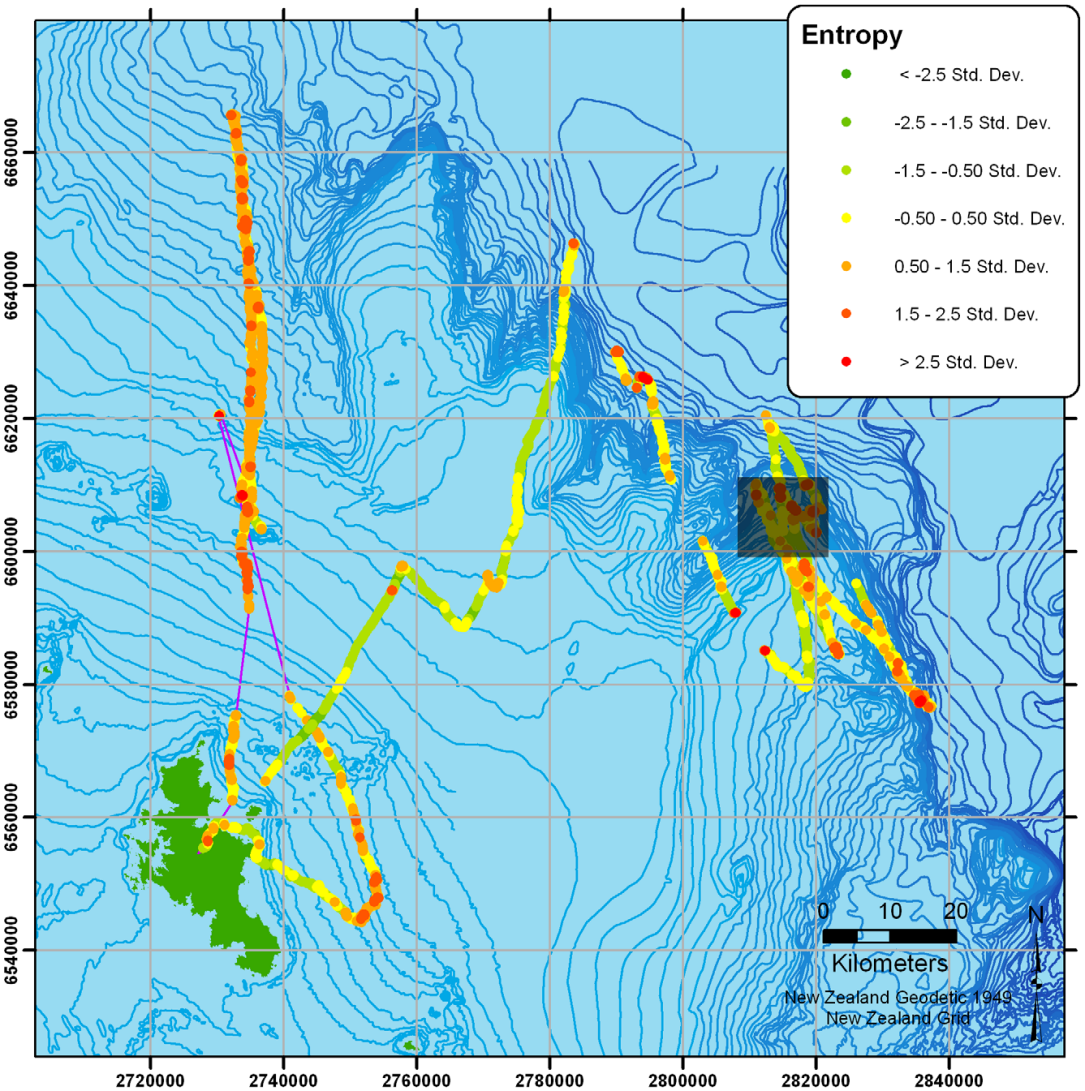
High-resolution tracks overlaid with spatio-temporal uncertainty (entropy) calculated at each location. Colours are categorised by the distribution of entropy along the track. Highlighted section is shown in figure 9.

**Figure 9:**
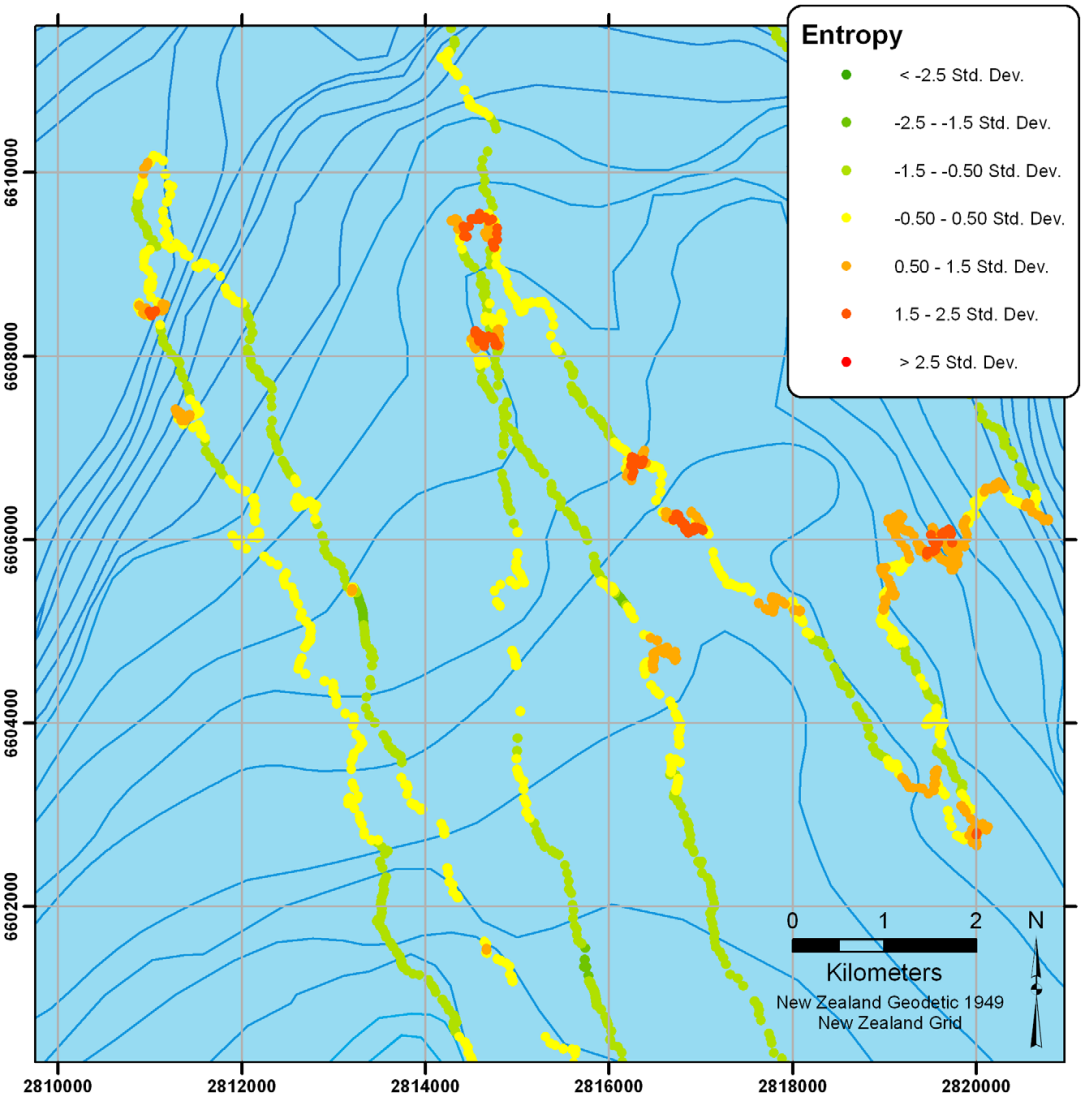
Zoomed section from figure 8 showing a high-resolution track overlaid with spatio-temporal uncertainty (entropy) calculated at each location. Colours are categorised according to the distribution of entropy along the track and show regions of high-predictability (low-entropy) in green contrasting with sections of highly unpredictable movement (high-entropy) in red.

Figure 8 shows the two high-resolution tracks overlaid with a measure of path predictability or tortuosity at each location (measured here using spatio-temporal entropy: see [15], [16] for details). The right hand-bird can be seen heading out the shelf-break (or slope) where it sweeps back and forth in 30–70 km sections. In both birds, high-entropy (highly unpredictable) regions can be seen throughout the tracks. Figure 9 presents a closer view of the highlighted section in figure 8, which shows the high-entropy sections at local turns.

## Discussion

This study represents the first attempt to track with precision the foraging excursions of the New Zealand endemic Black Petrel, *Procellaria parkinsoni*. Its vulnerability and relatively small size (700 g) demanded caution, and therefore the data set is inevitably small. Nevertheless, a consistent snapshot emerges of the black petrel's behaviour at sea. Although we were unable to compare directly with control individuals, all tracked birds returned, and at approximately similar mass before and after deployment (where measured). Furthermore, returning birds were generally heard to feed their chick before recovery of the tracking devices. Hence, it is reasonable to infer that tracked behaviour at sea was indicative of successful foraging.

Most striking is the observation that there appear to be dual foraging strategies. First, most logged trips showed that birds fly more or less directly to apparent foraging grounds above the edge of the continental shelf. These trips varied in distance from the colony, but the relationship with bathymetry is reasonably consistent. The importance of the shelf-break (or slope) is particularly noticeable when birds are moving slowly and relatively near the colony (figures 5 and 6), where locations peak in regions of 600–1000 m depth (highlighted in figure 7). Shelf-breaks are commonly considered important for foraging procellariforms [6], [25], because associated upwellings often cause high productivity and may be signalled by high levels of dimethyl sulphide [26]. There is some evidence in the tracks that birds may be utilising different areas along the shelf, with concentrations of activity immediately northeast of the colony, and longer trips southeast to the waters off East Cape. Sample size is too small to confirm that these areas are of particular importance, but the data show that birds are not simply concentrating their foraging efforts on that part of the shelf-break closest to the colony. In addition to foraging more locally on the shelf-break, the tracks indicate a second strategy involving much longer excursions. One bird flew more than 1100 km from the colony, another over 550 km, to regions not associated with the shelf-break and in water of depths of more than 2000 m. Again, such dual strategies have been found in other procellariforms [27]. We also examined the possible relationship between productivity in terms of concentration of chlorophyll *a*, but found no significant relationship.

There were two distinct peaks within the identified modes of movement, one corresponding probably to sitting and drifting with the current (mean speed 0.83 ms^−1^), the other to flying (mean speed 10.2 ms^−1^). The high variance within the fast flight mode was likely associated with whether birds were flying into or with the wind. The decomposition of behaviour into two modes using the Gaussian mixture model allowed us to identify the relationship between slow movement, into which feeding bouts are most likely to be classified, and the 600–1000 m depth range associated with the shelf-break. When birds were in flight there was no such association with depth.

In the two high-resolution tracks, birds were also more likely to be found over the shelf-break when sitting (rather than flying), suggesting a distinct commuting component. Nevertheless, one track in particular shows (figures 8 & 9) large-scale sweeping movements (the longest movement leg is 75 km long), often in flight, broadly parallel to the shelf-break. These patterns strongly suggest that the bird was patrolling the oceanic side of the shelf-edge back and forth for food, or signs therefore, on a scale that would not be obvious from traditional boat sightings (see also [28]). Closer examination of these sweeps shows that the roughly linear sections of tracks are punctuated with more tortuous regions of flight which may be indicative of locally restricted search behaviour. Whilst the current sample is small, it does indicate the potential for resolving detailed behaviour at sea from high-resolution location data alone.

More data will be required to provide a comprehensive understanding of the oceanic locations on which the Black Petrel's survival depends. Nevertheless, the current data show the importance of the oceanic shelf-break east of New Zealand, and of more distant oceanic forays, during this most sensitive period of the species' breeding cycle.

## Acknowledgments

This work would not have been possible without the support of NZ Department of Conservation, particularly Jo Sim and Halema Jamieson, on GBI, the help and advice of Claudia Duncan and Mark Fraser of Wildlife Management International Limited and the collaborative assistance of NIWA, Microsoft Research, Cambridge and Merton College, Oxford. All work was conducted in accordance with Oxford University's Local Ethical Review Procedures, and with the appropriate NZ licences and permissions.
